# Recurrent Postmyocardial Infarction Ventricular Tachycardia: An Unusual Culprit

**DOI:** 10.1155/2015/564394

**Published:** 2015-11-22

**Authors:** Akhil Kumar Sharma, Nirdesh Jain, Safal Safal, Vikas Kumar, Sudhanshu Kumar Dwivedi

**Affiliations:** Department of Cardiology, King George's Medical University, Lucknow 226003, India

## Abstract

Although temporary transvenous pacing is life-saving in patients with myocardial infarction who develop bradyarrhythmias, the electrical complications resulting from it can be fatal and are rarely reported. We report here a patient with acute inferior wall myocardial infarction who required temporary transvenous pacing due to second-degree atrioventricular block accompanied with hypotension. Following coronary angiography and successful revascularisation, the patient developed multiple episodes of monomorphic and polymorphic ventricular tachycardia as well as ventricular fibrillation which on careful inspection were found to be initiated by fusion of the intrinsic and paced complexes. The problem of malignant ventricular tachycardia was solved by simple removal of the pacing lead. To the best of our knowledge, malignant ventricular tachycardia of both monomorphic and polymorphic types initiated by fusion complexes in a paced patient has not been reported in literature.

## 1. Introduction

Temporary transvenous pacing is a life-saving intervention in many situations, most commonly in complete heart block, whatever the cause may be. The situation is more dramatic when heart block is associated with myocardial infarction (MI). Although a relatively safe procedure, mechanical complications resulting from temporary transvenous pacing, like lead perforation, cardiac tamponade, pneumothorax, and hemothorax, are well known. However, sustained ventricular tachycardias (VT) due to temporary pacing are uncommonly reported [1–4]. Those that are reported are frequently explained by an “R-on-T” phenomenon due to abnormal sensing of the artificial pacemaker. This pacing lead induced VT is mostly (80%) of polymorphic type [2]. Here we report an unusual case of monomorphic and polymorphic ventricular tachycardia/fibrillation that were caused by temporary pacemaker induced fusion beats in a patient with inferior wall myocardial infarction.

## 2. Case Report

**Figure 1:**
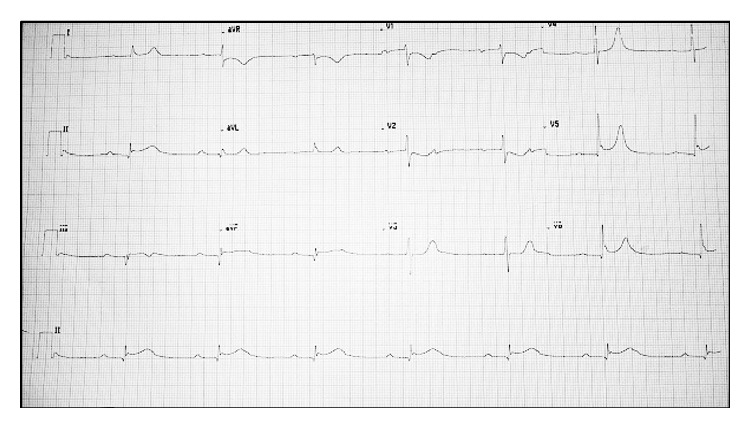
Baseline ECG of the patient, showing ST coving in inferior leads {II, III, and aVF}, and V_5-6_ suggestive of inferior wall MI along with 2 : 1 atrioventricular block.

A 65-year-old nondiabetic male presented to us with history of chest pain of 3-hour duration two days ago, associated with vomiting and one episode of syncope. For these complaints, he had been evaluated at a local hospital and diagnosed as acute inferior wall with posterior wall MI with atrioventricular (AV) dissociation, for which he was managed medically, and transvenous right ventricular apical temporary pacing was done. Due to persistent hypotension and recurrent rest pain, the patient was referred to us on the third day of the index event. His presentation ECG at our centre showed evidence of inferior wall MI with 2 : 1 AV block (Figure 1). Echocardiogram showed hypokinesia in the left circumflex (LCx) territory, with an ejection fraction of 50% and moderate mitral regurgitation. Pacing threshold was normal and remained so throughout the observation period. In view of postinfarction angina, hypotension, and persistent AV block, the patient was taken up for coronary angiography with an intent to revascularise. His coronary angiography showed a left dominant circulation with 90% stenosis in proximal segment of left circumflex artery and 70% stenosis in the middle segment of the left anterior descending (LAD) artery. The LCx was balloon dilated followed by deployment of a drug eluting stent; the LAD was planned for a staged procedure. The angioplasty was uneventful and the patient was shifted to ICCU.

**Figure 2:**
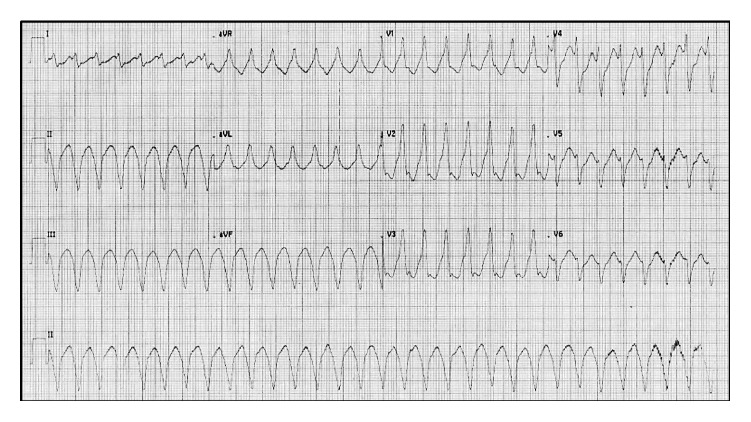
Monomorphic VT during hospital stay on day 4 of MI. It occurred after successful revascularisation without any evidence of ongoing ischemia or metabolic derangements.

**Figure 3:**
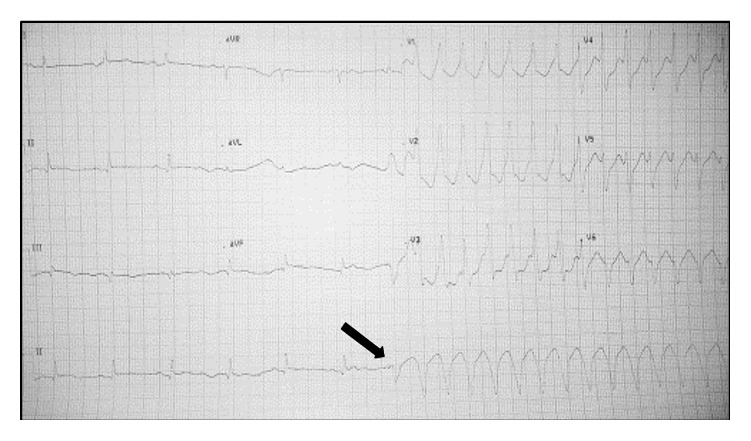
Monomorphic VT clearly appears to be starting with a fusion complex of the paced and intrinsic beats (indicated by arrow).

**Figure 4:**
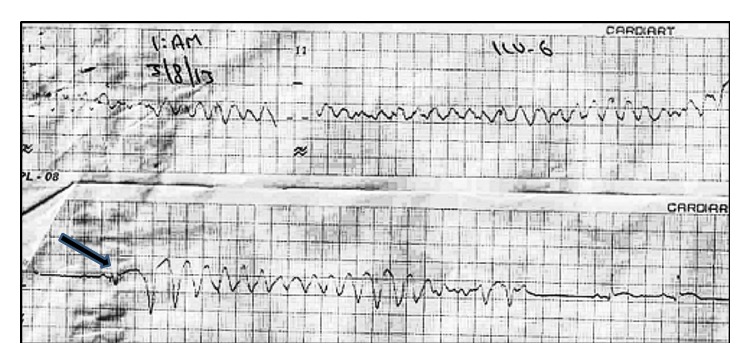
Same patient having polymorphic VT. Note the fusion complex prior to VT initiation.

After four hours of percutaneous coronary intervention (PCI), the patient suddenly developed palpitations and ECG showed monomorphic ventricular tachycardia (Figure 2). He was managed with 150 J biphasic DC cardioversion followed by intravenous amiodarone bolus and infusion, considering it to be secondary VT. The serum electrolytes were within normal limits. There were multiple hemodynamically significant episodes of both monomorphic (Figure 3) and polymorphic VT (Figure 4) over the next few hours. Significant ischemia was effectively ruled out by a check angiography that showed patent LCx stent with TIMI 3 flow as well as an insignificant rise in CPK-MB.

Careful inspection of serial ECG tracings of the patient showed that each episode of either monomorphic or polymorphic VT began after a fusion complex consisting of the paced and intrinsic beats (Figures 3 and 4). The temporary pacing lead was removed and there were no VT episodes after that. The patient had an uneventful convalescence; he was taken off amiodarone and discharged in a hemodynamically stable condition few days later. Staged PCI to LAD was done one month later; the procedure was uneventful. The patient is currently in our follow-up and doing well.

## 3. Discussion

Pacing lead induced VT is most commonly encountered during lead manipulation, when it can clearly be said to be anticipated. However, there are scanty articles which discuss lead induced VT that occurs after a pacemaker has been successfully implanted. Himmrich et al. [2] considered an episode of VT to be pacemaker induced if the onset was after a single visible and effective pacemaker stimulus. Of the mechanisms proposed to explain pacemaker induced VT, the “R-on-T” phenomenon is the most commonly put forward one, where a sensing defect causes the pacemaker to fire in the vulnerable period of the cardiac cycle, precipitating a VT/VF episode. Up to 80% of such cases are polymorphic VT.

However, in our case the “R-on-T” phenomenon is not seen. Instead, each episode can be seen arising after a fusion complex that consists of both the paced and intrinsic R waves. Lefroy et al. [5] have reported a case of recurrent VT in a patient with a permanent pacemaker that was consistently seen to arise from fusion of a paced beat and a ventricular extrasystole. They proposed that while each on its own was incapable of triggering a VT, the fusion complex had a greater propensity to do so. In our case, it is important to note that the pacemaker lead was already situated in a vulnerable zone either within or in close proximity to infarcted and electrically unstable myocardium. While a paced beat on its own did not trigger a VT, as during threshold testing, a fusion of the intrinsic and paced beats repeatedly did so. The electrical heterogeneity of the myocardium, with differential refractoriness along the rim of the infarcted territory as well as dead tissue at centre of the infarct, possibly explains both monomorphic and polymorphic VT arising from the same trigger at different times.

This case serves as an important learning point on two counts; first, one should be judicious while taking a decision to implant a temporary pacemaker in a post-MI situation and secondly, pacemaker induced VT must be high on one's checklist when confronted with VT in a patient with a pacemaker. After all, we would not want to implant an ICD where lead removal or readjustment is all that is warranted.
